# An independently validated, portable algorithm for the rapid identification of COPD patients using electronic health records

**DOI:** 10.1038/s41598-021-98719-w

**Published:** 2021-10-07

**Authors:** Su H. Chu, Emily S. Wan, Michael H. Cho, Sergey Goryachev, Vivian Gainer, James Linneman, Erica J. Scotty, Scott J. Hebbring, Shawn Murphy, Jessica Lasky-Su, Scott T. Weiss, Jordan W. Smoller, Elizabeth Karlson

**Affiliations:** 1 Channing Division of Network Medicine, Department of Medicine, Brigham and Women’s Hospital, 181 Longwood Ave, Boston, MA 02115 USA; 2 Section of Clinical Sciences, Division of Rheumatology, Brigham and Women’s Hospital, 75 Francis Street, Boston, MA 02115 USA; 3 Department of Medicine, Harvard Medical School, Boston, MA USA; 4 VA Boston Healthcare System, Jamaica Plain, MA USA; 5 Research Information Science and Computing, Mass General Brigham (Formerly Partners Healthcare Systems), Somerville, MA USA; 6 Office of Research Computing and Analytics, Marshfield Clinic Research Institute, Marshfield, WI USA; 7 Department of Neurology, Massachusetts General Hospital, Boston, MA USA; 8 Department of Psychiatry, Massachusetts General Hospital, Boston, MA USA; 9 Psychiatric and Neurodevelopmental Genetics Unit, Center for Genomic Medicine, Massachusetts General Hospital, Boston, MA USA

**Keywords:** High-throughput screening, Machine learning, Predictive medicine, Epidemiology, Population screening, Medical research

## Abstract

Electronic health records (EHR) provide an unprecedented opportunity to conduct large, cost-efficient, population-based studies. However, the studies of heterogeneous diseases, such as chronic obstructive pulmonary disease (COPD), often require labor-intensive clinical review and testing, limiting widespread use of these important resources. To develop a generalizable and efficient method for accurate identification of large COPD cohorts in EHRs, a COPD datamart was developed from 3420 participants meeting inclusion criteria in the Mass General Brigham Biobank. Training and test sets were selected and labeled with gold-standard COPD classifications obtained from chart review by pulmonologists. Multiple classes of algorithms were built utilizing both structured (e.g. ICD codes) and unstructured (e.g. medical notes) data via elastic net regression. Models explicitly including and excluding spirometry features were compared. External validation of the final algorithm was conducted in an independent biobank with a different EHR system. The final COPD classification model demonstrated excellent positive predictive value (PPV; 91.7%), sensitivity (71.7%), and specificity (94.4%). This algorithm performed well not only within the MGBB, but also demonstrated similar or improved classification performance in an independent biobank (PPV 93.5%, sensitivity 61.4%, specificity 90%). Ancillary comparisons showed that the classification model built including a binary feature for FEV1/FVC produced substantially higher sensitivity than those excluding. This study fills a gap in COPD research involving population-based EHRs, providing an important resource for the rapid, automated classification of COPD cases that is both cost-efficient and requires minimal information from unstructured medical records.

## Introduction

The estimated world prevalence of COPD ranges from 4–10%, and it is projected to be the third leading cause of mortality by 2030^1,2^. Diagnosis of COPD can be confirmed through spirometry demonstrating a forced expiratory volume in 1 s (FEV_1_) to forced vital capacity (FVC) ratio of < 0.7 that persists after administration of inhaled bronchodilators^3^. However, relying solely on FEV_1_/FVC to diagnose patients in clinical settings is marked by both under- and misdiagnoses^4–6^, as it comprises only one parameter of the criteria necessary to establish COPD. In most epidemiological studies of COPD, ascertainment of case populations thus frequently requires labor-intensive, direct medical review in addition to testing (e.g. spirometry) by trained clinicians. This process is a significant rate-limiting factor in large-scale COPD studies, especially in the development of new cohorts.

Electronic health record (EHR) databases and linked biorepositories have become increasingly common^7–9^, and present a major opportunity for disease research. Numerous studies have demonstrated the utility of leveraging EHRs and International Classification of Diseases (ICD) codes for the accurate identification of complex, heterogeneous conditions such as asthma, rheumatoid arthritis, bipolar disorder, and Alzheimer’s disease^10–14^. The development of EHR algorithms can facilitate rapid, low-cost, and large population-based explorations of disease that can be employed across a variety of studies, such as pharmacologic surveillance^15–17^, personalized medicine^18,19^, and genetic association^9,10,20^.

Prior EHR-based classification heuristics and algorithms for COPD have reported low positive predictive value (PPV) estimates ranging from 36.7% to 80.7%^21–23^. Therefore, our aim was to develop a high-performing, portable COPD classification algorithm using EHR data in the Mass General Brigham Biobank (MGBB). This diagnostic classification algorithm facilitates rapid identification (i.e., without requiring labor-intensive manual chart reviews by clinicians) of COPD cases with high specificity. It has been independently validated, and has been successfully implemented across a larger national consortium of biobanks called the Electronic Medical Records and Genomics (eMERGE) network.

## Methods

Study participants were drawn from the Mass General Brigham (MGB; Boston, MA; formerly Partners Healthcare Systems) Biobank, which is a subset of the MGB Research Patient Data Registry (RPDR). The RPDR is a data warehouse that gathers data from multiple hospital electronic record systems within MGB and includes over 4.6 million patients from MGB hospitals, 227 million encounters, and 2.4 billion distinct, coded clinical facts dating back to 1986 including demographic data, diagnostic codes, procedures, pharmacy data (e.g. RxNorm), inpatient and outpatient encounter information, laboratory data, imaging/pathology data. The MGBB is a collection of DNA, serum, and plasma samples from participants (recruitment ongoing) from MGB hospitals who provided informed consent for a) broad use for genomics, biomarker, epidemiology research, and b) linkage with RPDR EHRs and survey data. At the time of analysis, the MGBB comprised approximately 77,000 consented participants. Demographics of the MGBB are broadly representative of demographic composition of Eastern MA. Minority enrollment reflects population demographics (13% non-White).

**Figure 1:**
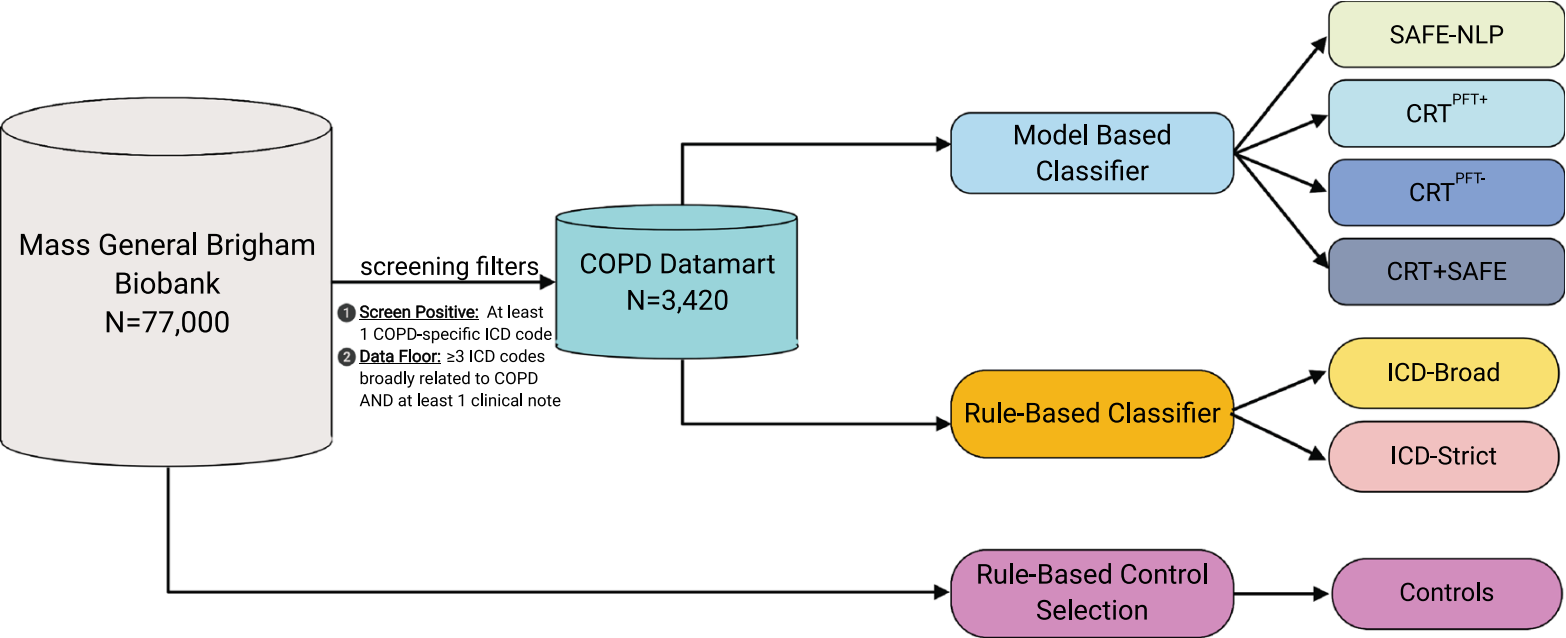
Overview of COPD datamart selection and developed algorithms.

**Figure 2:**
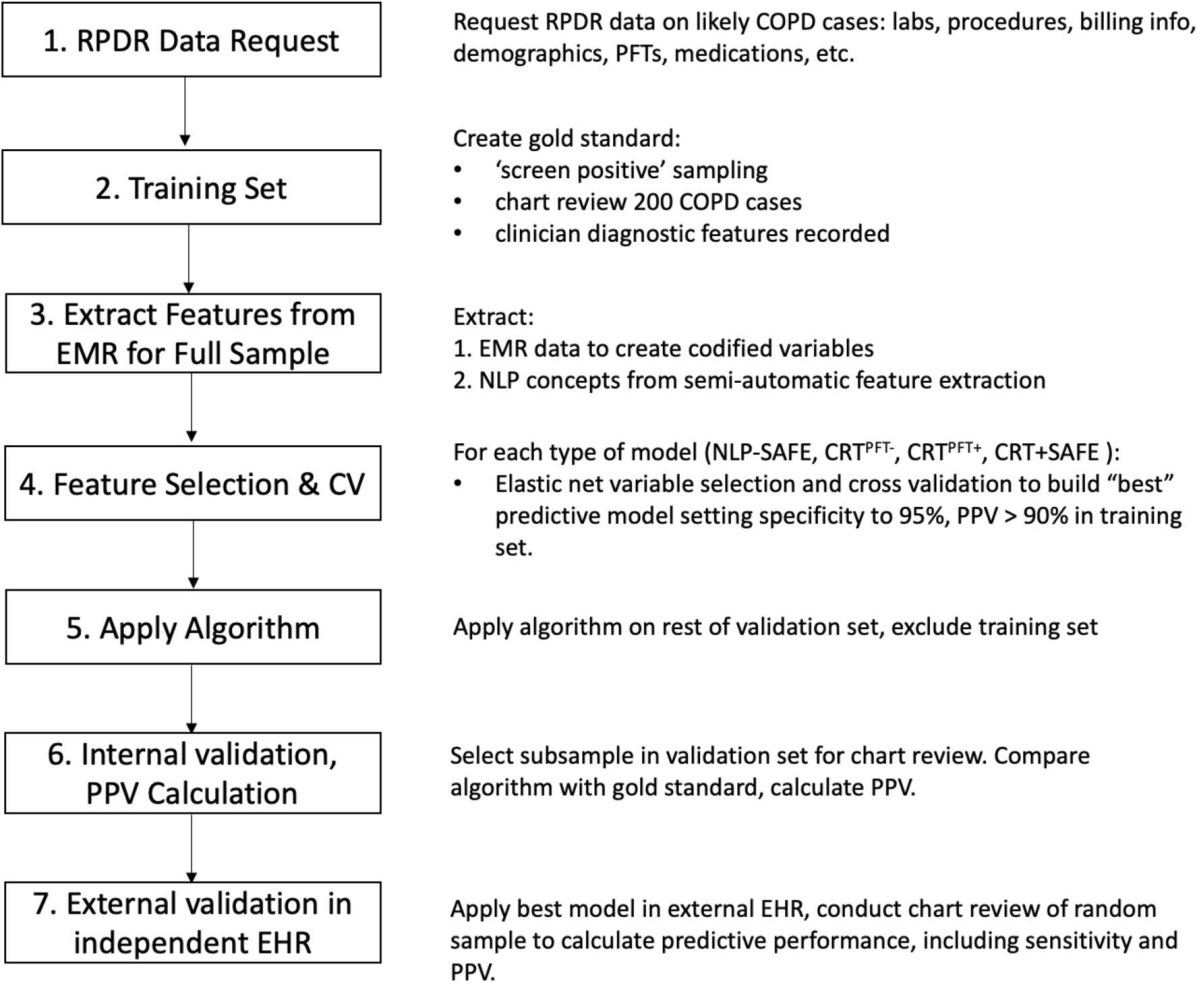
Broad overview of steps in phenotyping algorithm development.

Patients with one International Classification of Diseases (ICD) code *specific* to COPD (ICD9: 491.2 obstructive chronic bronchitis, 493.2 chronic obstructive asthma, 496.* chronic airway obstruction, not elsewhere classified ; ICD10: J43* emphysema, J44* other chronic obstructive pulmonary disease) were selected to generate a “*screen positive*” COPD datamart. Next, we implemented a *data floor* threshold whereby subjects with 3 ICD codes *broadly* associated with COPD (ICD9: 491.* chronic bronchitis, 492.* emphysema, 493.2 chronic obstructive asthma, 496.* chronic obstructive pulmonary disease, not elsewhere classfiied; ICD10: J40* bronchitis, not specified as acute or chronic, J41* simple and mucopurulent chronic bronchitis, J42* unspecified chronic bronchitis, J43* emphysema, J44* other chronic obstructive pulmonary disease) occurring on distinct dates, and at least one unstructured medical note (i.e., narrative text from patient health records regarding reason for visit, discharge, operation, labs, etc.) in their records, were selected from the COPD datamart to create our patient chart review pool. This was to ensure that study subjects had sufficient EHR and medical record histories to be informative in the algorithm development process. An overview of these filtering steps can be seen in Fig. 1, and an overview of general model development can be seen in Fig. 2. The protocol for this study was approved by the Brigham and Women’s Hospital and the Mass General Brigham institutional review boards, #2015P000983. All methods were performed in accordance with relevant guidelines and regulations.

### Clinician chart reviews

A random sample of 200 participants was selected from the COPD screen positive set, among whom 182 met the data floor selection criteria for chart review. Chart reviews were performed by two senior pulmonologists (M.H.C. and E.W.) to establish a gold standard training set using an internally developed chart review protocol (Figure S2). To ensure acceptable interrater reliability, 25 charts were reviewed by both pulmonologists and consistency was assessed using Cohen’s kappa statistic. Charts with available pulmonary function tests (PFTs) such as spirometry reports within the past 10 years were reviewed for the presence of values of FEV1/FVC < 0.7 to classify ‘definite’ COPD cases. Charts without available PFT reports were reviewed for clinical COPD criteria based on ≥ 3 of the following: (1) ever smoking, (2) ≥ 2 notes confirming a clinical COPD diagnosis, (3) moderate or severe centrilobular or panacinar emphysema on clinical chest computed tomography (CT) scan, (4) COPD-specific medications or (5) presence of pulmonologist-diagnosed COPD. Reviewers classified patients meeting these criteria but lacking confirmatory spirometry as ‘clinical’ COPD cases. Finally, for internal validation, 100 participants with charts were randomly selected and reviewed from the screen-positive set to comprise a gold-standard test set.

### Rule-based algorithms

Algorithms relying strictly on ICD codes to identify COPD cases have previously been proposed and applied^22^. Therefore, as a performance benchmark for our algorithm, we employed rule-based heuristics to classify COPD cases based only on ICD codes among the screen positive set: (1) *ICD-strict:* 3 COPD-specific ICD codes, and (2) *ICD-broad:* 2 COPD-specific codes, where each code was required to have occurred on distinct dates in the EHR for a given patient. A rule-based algorithm was also applied to identify controls (i.e. subjects with no history of COPD-related codes), requiring 0 COPD-specific codes and 2 encounters in the EHR.

### Model-based algorithms

To develop model-based algorithms, we first constructed a comprehensive feature space from which predictive variables of COPD case status could be selected. Variables were derived from (1) structured data such as ICD, current procedural terminology, and prescription codes, and (2) unstructured data, such as narrative text present in clinical visit and discharge notes or radiology reports. Coded features derived from the structured data were obtained based on COPD-related risk factors partly curated by the two chart reviewers or drawn from prior literature^21^. These included derived variables relating to age at first COPD diagnosis, smoking history, medical utilization, PFTs, visit history to pulmonary clinics, ICD codes, COPD and asthma medications, and radiography. Values for FEV1, FVC, and smoking status were obtained from spirometry reports using NLP algorithms in R (Supplementary Information 5 and 6), and all other NLP variables were constructed using surrogate assisted feature extraction (SAFE)^24^, which has been described in detail previously^25^. Briefly, the implementation of the SAFE procedure proceeds as follows: (1) extraction of United Medical Language System (UMLS) concepts related to COPD from publicly available databases including Medscape, PubMed, and Wikipedia via named entity recognition, (2) application of Narrative Information Linear Extraction^25^, to scan EHR clinical narratives for positive mentions of the UMLS terms, which are then totaled to obtain patient-level counts, (3) feature selection based on majority voting (concept present in the majority of databases scanned), frequency control (concept present in > 5% medical notes mentioning COPD), and surrogate selection criteria (concept selection based on predictiveness of COPD ICD counts and primary NLP terms).

After creating the feature space, adaptive elastic net regularization was applied to build logistic regression classifiers of COPD status using the R package glmnet. Tuning parameters, for the regularization penalty and for the mixing parameter between ridge () and lasso () regression, for the elastic net were selected through fivefold cross validation using the package caret in R^26^.

COPD status from chart reviews was classified as a binary variable combining ‘definite’ and ‘clinical’ into COPD versus non-COPD. Classification models were built using several different feature space combinations: (1) expert and literature curated features excluding spirometry derived variables (CRT^PFT-^), (2) expert and literature curated features including spirometry derived variables (CRT^PFT+^), (3) SAFE-extracted features only (SAFE-NLP), and (4) combined CRT^PFT+^ and SAFE features (CRT + SAFE). For each model, the set of features most predictive of COPD in the gold-standard training set were identified, and the relative weights (beta coefficients) of the features were extracted. After feature selection, classification thresholds were selected holding the specificity level at ~ 95%, where those above the cutoff were assigned case status, and those below were assigned non-case status. The model with the best performance in terms of 1) total area under the curve (AUC) of the receiver operator characteristic curve and 2) estimated sensitivity, positive predictive value (PPV), and negative predictive value (NPV) at the 95% specificity threshold was taken forward for algorithm validation. Confidence intervals for performance estimates were obtained via bootstrap. F1 and F0.5 metrics of performance were also calculated for the final model. An overview of the different criteria for all model and rule-based algorithms can be seen in Table 1.

**Table 1:** Algorithms for classifying chronic obstructive pulmonary disease.

Classification method	Classifier description	Minimum selection criteria		
		ICD9/10 Diagnosis criteria	Visit criteria	Other criteria
**Rule-based**				
ICD-strict^a^	3 COPD-specific codes	3 or more COPD-specific codes	None	
ICD-broad^b^	2 COPD-specific codes	2 or more COPD-specific codes	None	
Control selection	0 COPD-specific codes	Subjects with no history of COPD related codes	2 encounters in MGB Biobank	
**Model-based**^**c**^				
*Automatic extraction NLP features*				
SAFE-NLP	Model selected from surrogate assisted feature extraction with natural language processing of unstructured EHR data (narrative text from clinic notes)	At least 1 COPD-specific code and at least 3 broad COPD codes	1 visit with electronic clinical note in the EHR	Selected by classifier
*Curated (CRT) features*				
CRT^PFT-^	Model selected from literature-based and expert-curated feature inputs primarily derived from structured data, *excluding* measures of spirometric FEV_1_/FVC performance	At least 1 COPD-specific code and at least 3 broad COPD codes	1 visit with electronic clinical note in the EHR	Selected by classifier
CRT^PFT+^	Model selected from the feature space of CRT^PFT-^, but *inclusive* of measures of spirometric FEV_1_/FVC performance	At least 1 COPD-specific code and at least 3 broad COPD codes	1 visit with electronic clinical note in the EHR	Selected by classifier
*Mixed (automatic* + *curated) features*				
CRT + SAFE	Model based on combining the full feature space for CRT^PFT+^ and SAFE	At least 1 COPD-specific code and at least 3 broad COPD codes	1 visit with electronic clinical note in the EHR	Selected by classifier

^a^COPD-specific codes include: 1) ICD9: 491.2, 493.2, and 496.*; 2) ICD10: J43.* or J44.*.

^b^Broad COPD codes include any codes with the following base numbers: 1) ICD9: 491.*, 492.*, 493.2*, and 496.*; 2) ICD10: J40.*, J41.*, J42.*, J43.*, J44.*.

^c^All model-based algorithms were developed using probability-based thresholding via logistic regression models selected using a threshold for specificity at 95%.

### Internal and external validation

Internal validation to confirm algorithm performance was conducted in the gold standard test set in the MGBB. To assess the portability of the selected MGBB algorithm, external validation was performed at an independent site with a different EHR system: Marshfield Clinic Health System (MCHS; Marshfield, WI). MCHS is an integrated health care delivery system that provides the majority of healthcare services to 1.5 million patients residing in more than 50 locations in northern, central, and western Wisconsin. The MCHS has coded diagnoses dating back to the early 1960s, and employs a modern integrated, internally developed EHR and data warehouse beginning in the 1990s.

Using identical filtering procedures as in the MGBB, a COPD datamart was constructed in the Marshfield EHR. Among eligible subjects meeting the datamart screening criteria, a random set of 100 subject charts were selected for chart review using the clinical heuristic algorithm developed in the MGBB. After applying the MGBB COPD algorithm, sensitivity, specificity, PPV, NPV, and F1 and F0.5 metrics were calculated to assess algorithm performance in the Marshfield validation sample.

### Ethical approval

The eMERGE study protocol was approved by the institutional review board at Mass General Brigham (formerly Partners Healthcare System) and Marshfield Clinic Research Institute. All authors read and approved the manuscript.

## Results

The demographic characteristics of the randomly sampled gold standard training and test sets, as well as the full COPD datamart, can be seen in Supplementary Table 1. In total, 77 patients were classified with COPD, and 105 were defined as non-cases in the full gold-standard training set (N = 182), and 46 and 54 patients were classified as cases and non-cases in the gold standard test set (N = 100). Spirometric FEV_1_/FVC results were available for 129 (71%) patients in the training set, and 80 (80%) patients in the test set. Within a random set of 25 subject charts reviewed by both pulmonologists, high interrater reliability of COPD diagnosis was observed with an estimated Cohen’s kappa of 0.867 (p = 6.6 × 10^–9^).

We identified and derived 56 variables for consideration in the curated (CRT) feature space (Supplementary File: Code Book for Structured Data Features). Medications associated with pulmonary diseases were classified by specificity for COPD management (Supplementary Table 2). In total, 53 NLP features were identified by SAFE for use in model training to construct the SAFE-NLP and CRT + SAFE classifiers (Supplementary Table 3).

The two rule-based algorithms using the naïve ICD-based thresholding approach to classify COPD status performed similarly in the gold standard training set. The ICD-Strict algorithm had 98.1% sensitivity, 11.7% specificity, 60.2% PPV, and 81.8% NPV, with F1 of 0.746 and F0.5 of 0.652, and the ICD-Broad algorithm demonstrated 100% sensitivity, 3.9% specificity, 58.7% PPV and 100% NPV, with F1 = 0.740 and F0.5 = 0.877. As the specificity for both rule-based algorithms were extremely low, neither was taken forward for assessment in the MGBB test set (Table 2).

**Table 2:** Comparison of performance characteristics between different electronic medical record COPD classification algorithms within Mass General Brigham Biobank training set (N = 182).

Algorithm	Counts (N)				Algorithm Performance (95% CI)*			
	True positive	True negative	False positive	False negative	Sensitivity	Specificity	PPV	NPV
**Rule-based**								
ICD-Strict	103	9	68	2	0.981	0.117	0.602	0.818
ICD-Broad	105	3	74	0	1	0.039	0.587	1
**Automatic NLP features**								
SAFE-NLP	38	73	4	66	0.365 (0.270–0.462)	0.948 (0.896–0.987)	0.905 (0.816–0.977)	0.525 (0.490–0.567)
**Curated features**								
CRT^PFT-^	42	72	5	63	0.400 (0.314–0.495)	0.935 (0.883–0.987)	0.894 (0.808–0.976)	0.533 (0.493–0.578)
CRT^PFT+^	62	73	4	43	0.590 (0.495–0.676)	0.948 (0.896–0.987)	0.939 (0.878–0.987)	0.629 (0.577–0.688)
**Mixed features**								
CRT + SAFE	47	73	4	62	0.404 (0.317–0.490)	0.948 (0.896–0.987)	0.913 (0.830–0.979)	0.541 (0.503–0.583)

^a^COPD-specific codes include: 1) ICD9: 491.2, 493.2, and 496; 2) ICD10: J43 or J44.

^b^Broad COPD codes include any codes with the following base numbers: 1) ICD9: 491, 492, 493.2, and 496; 2) ICD10: J40, J41, J42, J43, J44.

*All probabilistic algorithms were assessed at their corresponding thresholds specifying 95% specificity.

SAFE-NLP: Model based on surrogate assisted feature extraction with natural language processing of unstructured EHR data (free text); CRT^PFT-^: Model based on literature and expert-curated feature inputs primarily derived from structured data, excluding feature weights for spirometric FEV_1_/FVC performance; CRT^PFT+^: Model based on feature space of CRT^PFT-^, but inclusive of feature weights for spirometric FEV_1_/FVC performance; CRT + SAFE: Model based on combining the full feature space for CRT^PFT+^ and SAFE.

**Figure 3:**
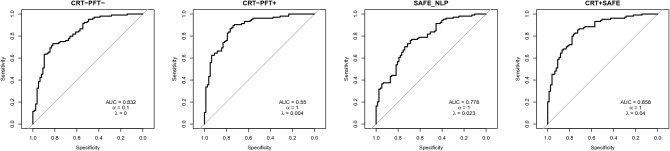
Receiver-operator characteristic curves to assess classification performance of model-based algorithms.

As COPD is not an uncommon disease, we prioritized optimizing specificity (i.e. reducing the false positive rate) over sensitivity (i.e. detecting all true positives) in the model-based algorithms. Among the four model-based algorithms, CRT^PFT+^ produced the highest AUC of 0.879. AUCs for CRT^PFT-^, SAFE-NLP, and CRT + SAFE were 0.834, 0.778, and 0.865, respectively (Fig. 3).

Performance characteristics of the CRT^PFT+^ model with specificity held at 95% yielded a sensitivity of 59.0%, PPV of 93.9%, an NPV of 62.9%, F1 of 0.725, and F0.5 of 0.840 in the gold standard training set (Table 2). The performance of the CRT^PFT-^ algorithm excluding spirometric results for FEV_1_/FVC revealed a lower sensitivity of 40%, a PPV of 89.4% and NPV of 53.3%, F1 of 0.553 and F0.5 of 0.450. The SAFE-NLP algorithm had 36.5% sensitivity, 90.5% PPV, 52.5% NPV, 0.520 F1, and 0.414 F0.5, while the CRT + SAFE algorithm had 40.4% sensitivity, 91.3% PPV, 54.1% NPV, F1 of 0.560, and F0.5 of 0.455. The model demonstrating the greatest overall AUC, as well as the highest PPV and sensitivity, was the CRT^PFT+^ model.

In the MGBB gold standard test set (N = 100), the CRT^PFT+^ algorithm had sensitivity of 71.7%, specificity of 94.4%, PPV of 91.7%, and NPV of 79.7% (Table 3). The CRT^PFT-^ algorithm had lower sensitivity more consistent with performance in the training set at 43.5%, 94.4% specificity, 87% PPV, and 66.2% NPV. The CRT + SAFE algorithm demonstrated 43.5% sensitivity, 98.1% specificity, 95.2% PPV, and 67.1%NPV. The SAFE-NLP algorithm had the lowest sensitivity of 37%, specificity of 94.4%, PPV of 85% and NPV of 63.8%. As the CRT^PFT+^ algorithm demonstrated comparably high PPV and higher sensitivity in the test set relative to its performance in the gold standard training set, the CRT^PFT+^ algorithm was taken forward for external validation in the Marshfield Clinic EHR (Table 3). Among the Marshfield screen positive set, chart reviewers classified 70 cases and 30 non-cases. Applying the CRT^PFT+^ model revealed independent validation performance of sensitivity 61.4%, specificity of 90%, PPV of 93.5%, NPV of 50% (Table 3) in the Marshfield validation sample.

**Table 3:** Comparison of performance characteristics of probabilistic electronic medical record COPD classification algorithms within Mass General Brigham Biobank validation set (N = 100) and external, independent validation of final algorithm in the Marshfield Clinic (N = 100).

Algorithm	Counts (N)				Algorithm Performance*			
	True positive	True negative	False positive	False negative	Sensitivity	Specificity	PPV	NPV
**MGBB Validation (N = 100)**								
Automatic NLP features								
SAFE-NLP	17	51	3	28	0.370	0.944	0.850	0.638
Curated features								
CRT^PFT-^	20	51	3	26	0.435	0.944	0.870	0.662
CRT^PFT+^	33	51	3	13	0.717	0.944	0.917	0.797
Mixed features								
CRT + SAFE	20	53	1	26	0.435	0.981	0.952	0.671
**External validation (N = 100)**								
Curated features								
CRT^PFT+^	43	27	3	27	0.614	0.900	0.935	0.500

*All probabilistic algorithms were assessed at their corresponding thresholds specifying 95% specificity.

*SAFE-NLP* Model based on surrogate assisted feature extraction with natural language processing of unstructured EHR data (free text), *CRT*^*PFT-*^ Model based on literature and expert-curated feature inputs primarily derived from structured data, excluding feature weights for spirometric FEV_1_/FVC performance, *CRT*^*PFT+*^ Model based on feature space of CRT^PFT-^, but inclusive of feature weights for spirometric FEV_1_/FVC performance, *CRT + SAFE* Model based on combining the full feature space for CRT^PFT+^ and SAFE.

Feature weight specifications for the CRT^PFT+^ algorithm are available in Table 4, and the alternative model weights are available in the supplement (Supplementary Table 4).

**Table 4:** Patient medical history features and weights used in the final Mass General Brigham Biobank CRT^PFT+^ algorithm for classification of COPD.

Model feature	Model weight	Variable type	Description
Intercept	− 1.871		
everPFTlt70	1.750	NLP	Ever had a pulmonary function test with spirometry indicating pre-bronchodilator FEV1/FVC ratio < 0.7 OR post-bronchodilator FEV1/FVC ratio < 0.7
nCOPDGTE3_365	0.465	Coded	Ever diagnosed with 3 or more COPD-related ICD codes within any rolling time window of 365 days
everTiotropium	0.334	Coded	Ever been prescribed tiotropium
iNotWhite	− 0.239	Coded	Race category denoting whether subject is White or Not White
smkEver	0.175	NLP	Any current/former history of smoking
everdxAtPulmClinic	0.056	Coded	Ever diagnosed with a COPD-related ICD code at a pulmonary clinic
everCOPDmed	0.048	Coded	Ever been prescribed a medication used to treat COPD?
nmedLAMA	0.017	Coded	Total count of distinct prescription codes for long acting muscarinic antagonists in participant medical record for treatment of lung diseases
pftCount	− 0.016	Coded	Total count of any kind of pulmonary function test
ageCOPDt1Specific	0.013	Coded	Age (in years) at first ICD code specific to COPD
nCOPD_ICD	0.013	Coded	The COPD feature count of distinct dates on which a subject has a code from this feature ICD10: J40–J44 ICD9: 491, 492, 493.2, 496
nBronchitis	− 0.009	Coded	The count of distinct dates on which a subject has a Bronchitis ICD code ICD10: J40, J41, J42 ICD9: 490, 491
nBronchiectasis	− 0.008	Coded	The count of distinct dates on which a subject has a Bronchiectasis ICD code ICD10: J47 ICD9: 494
patient_dxenct	− 0.001	Coded	Total number of encounters (visits) per subject with a coded diagnosis (any diagnosis not limited to COPD)

The case assignment threshold for this model, holding specificity at 95%, was 0.754. For subjects who were missing PFT results, the everPFTlt70 variable was classified as ‘No’ in this model.

## Discussion

In this study, we developed and compared several phenotyping algorithms for the classification of COPD in large EHR databases. We prioritized specificity and PPV for case selection in our algorithms, as the unbiased detection and estimation of associations between exposures and COPD is conditional on correct case classification. Among the models, we found that curated model CRT^PFT+^ performed with the greatest AUC, and also yielded the highest sensitivity and PPV while maintaining specificity of ~ 95%. The feature weight for everPFTlt70 was the largest weight in CRT^PFT+^, with several other clinical variables weighted highly including smoking history, having been diagnosed with a COPD-specific code 3 times in a rolling 365 day period, and having ever been prescribed tiotropium.

We also developed the CRT^PFT-^ model without inclusion of FEV_1_/FVC results from spirometry, as not all patients receive spirometric confirmation of COPD at diagnosis, particularly in primary care settings. This model demonstrated 19% reduced sensitivity and 4.5% reduced PPV relative to the CRT^PFT+^ model in the training set, and lower sensitivity by 28.2% and PPV by 4.7% in the test set (model weights available in Supplementary Table 4). Taken together, our findings are in line with what has been previously observed with respect to under- and mis-diagnosis trends for COPD in practice, where spirometry and clinical symptoms are both important features for appropriate diagnosis of COPD. Importantly, these findings demonstrate the inadequacy of relying solely on structured data features (e.g. counts of diagnostic codes) to identify high-confidence COPD cases in the EHR.

Strengths of this study include the large variety of features that were considered in the model building process, as the most essential step in developing prediction models is the curation of the feature space. We explored semi-automatic feature extraction approaches, in addition to manual development. In our study, we found that the use of SAFE did not perform as well as our expert curated models, and that combining the curated and SAFE-based feature spaces for model training did not appreciably improve the performance of the model, while making the algorithm significantly more laborious to apply. Therefore, we selected the CRT^PFT+^ model, which was largely based on derived variables from structured data in patient EHRs, with only two NLP-based variables for PFT and smoking (an overview of the steps for extracting the specific NLP variables in this model can be seen in Supplementary Information 5 and 6), to make the model more portable across institutions. More importantly, portability of the model was confirmed through external validation at the Marshfield Clinic which uses an entirely different home-grown EHR management system, with higher sensitivity (61.4%) reported than in our gold standard training set and an excellent PPV of 93.5%.

The advantage of employing an algorithm to classify COPD cases over manual chart review in a large EHR context is tremendous. Completing each chart reviews took an average of 15 min per patient in this study; manual chart review of the full MGBB COPD datamart (N = 3420) would have required 8 h/day of clinician labor for 107 days. Once the model terms are extracted from the EHR, our approach can be applied almost instantaneously and it is easily re-applied as new participants are enrolled or as EHR records update over time.

Aside from case identification for cohort development or population-based epidemiological research both within and across different institutions/biobanks, there may be more immediate potential clinical applications for the algorithms developed in this work. For example, the CRT^PFT-^ algorithm could be used to encourage providers to order PFTs among high-scoring patients. If employed as a pre-screening step, we note that probabilistic threshold indicating putative COPD could be relaxed to screen more subjects and thus improve overall capture of COPD cases, especially outside of specialty pulmonology clinics.

Our study is not without limitations. First, all of our models were developed using a COPD datamart built with a screening paradigm (i.e. multiple instances of *COPD-related* ICD codes, rather than multiple instances of *any* ICD codes) tailored for distinguishing between patients with COPD-like symptoms vs true COPD, which may have resulted in a less sensitive algorithm. However, an advantage of training on a more stringently screened sample is that the specificity for COPD is likely higher when applied to the general population, where we expect that distinguishing between COPD cases vs general controls and non-cases is less challenging than in our training set. In addition, the use of a screening step enhances PPV by increasing the prior probability of true cases in the sample to which the algorithm is applied. While none of the models performed outstandingly well with respect to NPV, our goal was to successfully make the more challenging distinction between COPD cases vs non-cases in the COPD datamart, rather than COPD cases vs controls in the general EHR; high confidence case ascertainment is essential for improving the power to detect even small associations between a given exposure and COPD (e.g. in molecular epidemiologic contexts). Thus, control selection was conceived as a rule-based approach requiring no history of any COPD-specific ICD codes, as has been successfully implemented previously^11,12^.

Second, our models are subject to the same vulnerabilities as any other predictive model in that that their development, and therefore their performance, depends significantly on the quality and source of the data inputs on which they are trained. In particular, while the demographic distribution of the MGB datamart was predominantly white, the randomly selected chart review sample from the external validation at Marshfield Clinic was entirely so; in both cases, the sampled participants were reflective of the demographic composition of the populations from which they were drawn. However, the consequence was that the predictive weight assigned to the indicator for White vs Not White was not applied in the execution of the algorithm at Marshfield and was not technically validated. Indeed, the weights assigned to this indicator variable in our curated models may reflect racial disparities in COPD diagnosis^27,28^ rather than a reliable predictor of true clinical COPD case status, and should thus be considered with appropriate caution in the absence of evidence to the contrary.

Finally, to apply our algorithm requires additional computational time to extract terms that are typically not reliably captured in structured EHR and are more often found in test results, lab reports, and clinical notes; however, we note that our final algorithm limits this work by restricting such extractions to just two features. On balance, the time saved by obviating more traditional, manual clinical review should still considerably outweigh the time required to extract even more extensive lists of NLP-based terms than the two proposed in our model.

In summary, we provide a high-performing, externally validated, and generalizable algorithm for the rapid classification of COPD using EHRs obviating the need for laborious manual chart review. Our algorithm relies primarily on structured data features, while minimizing dependency on unstructured records to just two features: smoking history and spirometry. In settings where support for text-mining of unstructured records is limited, we supply an alternative algorithm with high PPV as a viable alternative, albeit with lower sensitivity. While prior studies have used a variety of ICD-based strategies for identifying COPD patients, none have previously examined the impact of spirometry on classification performance. This study provides strong supportive evidence for the inclusion of spirometric features, in tandem with clinical features derived from structured records, in EHR investigations requiring classification of COPD.

## Supplementary Information

Supplementary Information.

## Data Availability

Data are available to investigators whose proposed use of the data are approved through an institutional review committee of Mass General Brigham.
