# Supplementary material for: An independently validated, portable algorithm for the rapid identification of COPD patients using electronic health records
Source: Sci Rep. 2021 Oct 7;11:19959. doi: 10.1038/s41598-021-98719-w (PMC8497529; doi:10.1038/s41598-021-98719-w)
Supplement: Supplementary file 1 — Supplementary Information. [file 41598_2021_98719_MOESM1_ESM.docx]

# **Gold Standard Training Set Chart Review Heuristic for COPD Classification**

**Diagnostician Review: COPD Chart Review Guide**

1. **Determine Diagnosis (see Figure S1):**
   - Yes (COPD):
     - Lung transplant AND pre-transplant diagnosis of COPD
       ***OR***
     - All of the following:
       - Age > 40
       - Confirmatory spirometry (either directly from PFT test results, or from text mentioning PFT test results in medical records):
         - Post-bronchodilator (BD) FEV1/FVC<0.7 if available; if not available, pre-BD FEV1/FVC<0.7
       - Absence of other primary diagnoses resulting in spirometric airflow obstruction (e.g. severe bronchiectasis, cystic fibrosis)
   - Yes (Clinical COPD):
     - Age > 40
       ***AND***
     - Spirometry (either directly from PFT test results, or from text mentioning PFT test results in medical records):
       - No spirometry
         **OR**
       - Spirometry taken >10 years ago, with pre- or post-BD FEV1/FVC>0.7

***AND***

- - - Three or more of the following:
      - Smoking History (Current or Former)
      - $\geq2$ ICDs for COPD from ICD list used in chart screening list (**Table 1**)
      - Moderate/severe/centrilobular/panacinar emphysema on clinical chest CT
      - COPD-specific medications
      - Pulmonologist diagnosis of COPD
  - No (Non-Case):
    - Age < 40

***OR***

- - - Normal, recent spirometry (Post-BD FEV1/FVC>0.7, or if not available, pre-BD and spirometry assessed <10 years ago)

***OR***

- - - Normal, old spirometry (Available pre-BD or post-BD FEV1/FVC>0.7 and spirometry assessed >10 years ago) **AND** fewer than three of the following:
      - Smoking History (Current or Former)
      - $\geq2$ ICDs for COPD from ICD list used in chart screening list (**Table 1**)
      - Moderate/severe/centrilobular/panacinar emphysema on clinical chest CT
      - COPD-specific medications
      - Pulmonologist diagnosis of COPD

**Figure S1. Broad overview of steps in phenotyping algorithm development.**

**
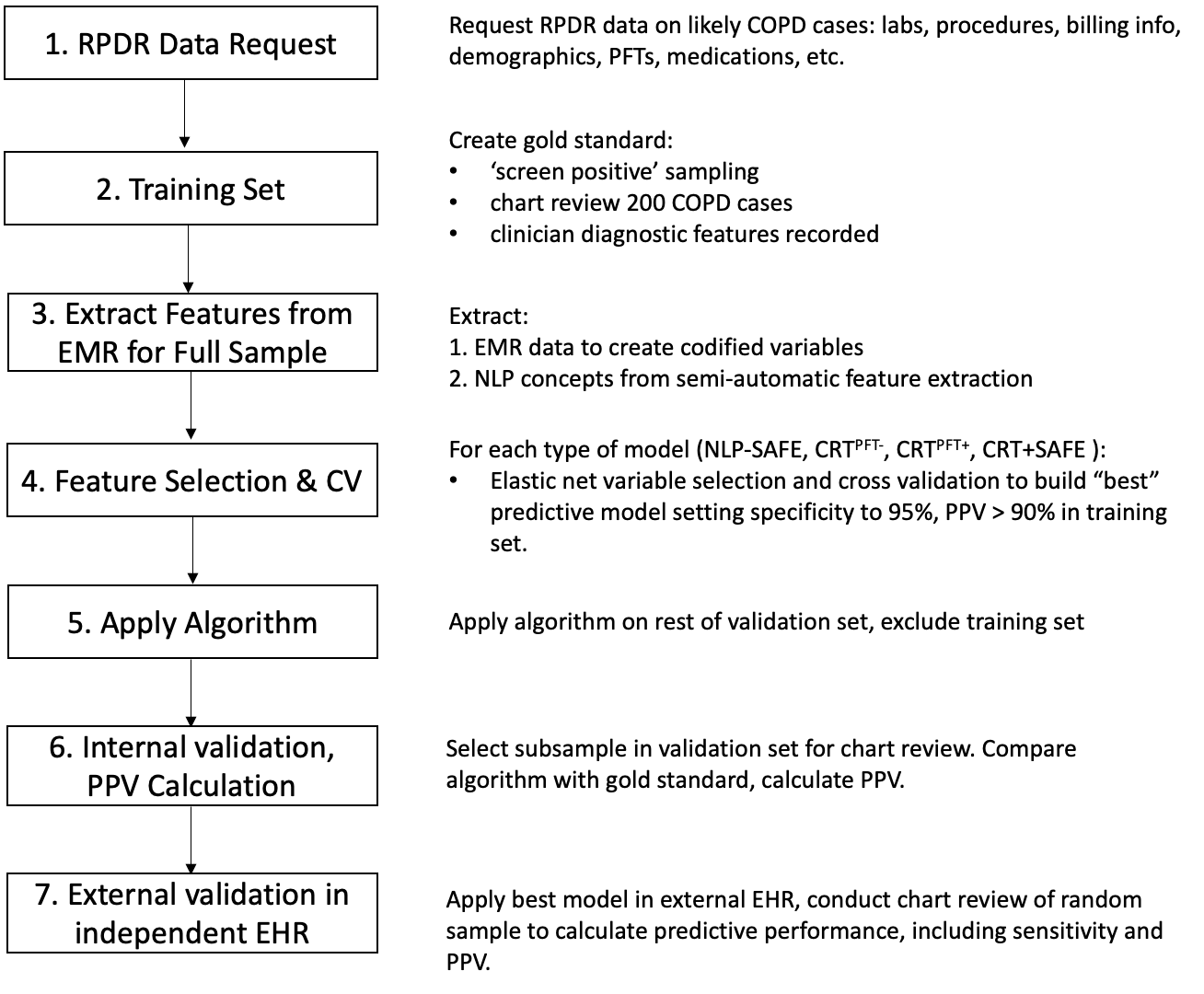
**

**Figure S1: Overview of COPD Classification Algorithm Gold Standard Chart Review Guidelines**


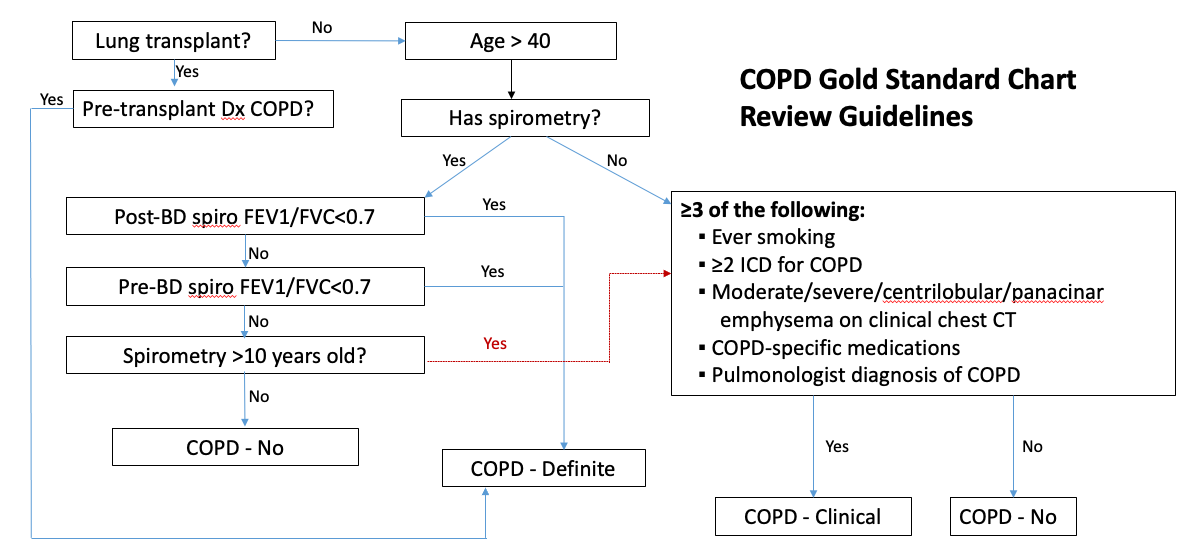


**Format for Diagnosis:**

- - Y (indicating Definite COPD Diagnosis)
  - Y (indicating Clinical COPD Diagnosis)
  - N (indicating No COPD)

# **Demographic Characteristics of MGBB Biobank COPD Datamart**

**Supplementary Table 1. Demographic characteristics of the Mass General Brigham Biobank COPD Datamart.**

| **Features** | **Training Set**  **(N=182)** | **Test Set**  **(N=100)** | **Full Datamart**  **(N=3420)** |
| --- | --- | --- | --- |
| Age in Years, Mean (SD) | 69.1 (11.2) | 71.7 (11.0) | 69.4 (11.6) |
| Female, N (%) | 97 (53.3) | 55 (55.0) | 1774 (51.9) |
| Race, N (%) |  |  |  |
| White | 156 (85.7) | 92 (92.0) | 3024 (88.4) |
| Ever Smoker, N (%) | 151 (82.0) | 83 (83.0) | 2791 (81.6) |
| Pulmonary Function Test in EHR, N (%)  Spirometric Testing, N (%) | 138 (75.8)  129 (70.9) | 85 (85.0)  80 (80.0) | 2352 (68.8)  2205 (64.5) |
| Chart Review Validated COPD Case, N (%) | 105 (57.7) | 46 (46.0) | - |

# **Classifications of Medications and Therapies for Obstructive Lung Diseases**

**Supplementary Table 2.** **Medications for obstructive lung disease classified by primary use application for the management of COPD, asthma, or both.** *Indicates medications which may also be used to treat severe asthma.

**
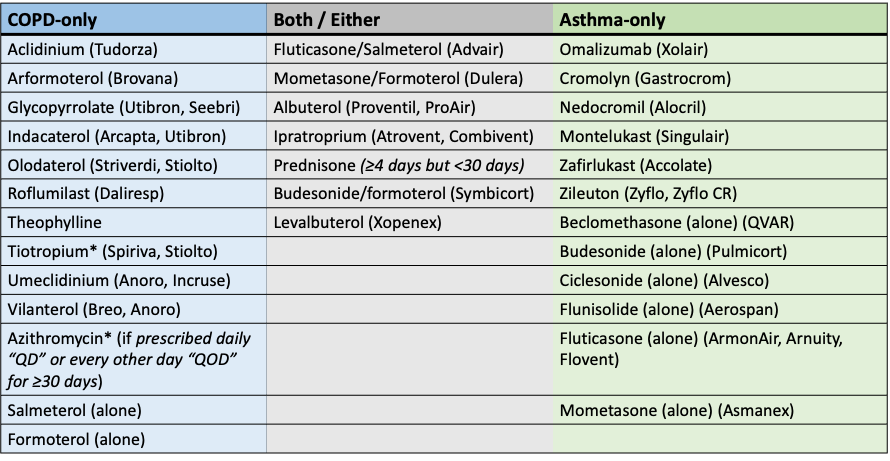
**

# **Full List of Expert and Literature Curated Features Extracted from the Structured Electronic Medical Record (EMR)**

For the full list of expert and literature curated features, definitions, and coding, please see additional file: Code Book for Structured Data Features.

# **Full List of Surrogate Assisted Feature Extraction (SAFE) Concept Unique Identifiers for COPD**

**Supplementary Table 3**

| **CUI** | **Term** |
| --- | --- |
| C0001617 | adrenal_cortical_hormone |
| C0003240 | macrolide_antibiotic |
| C0003385 | cholinergic_muscarinic_antagonist |
| C0004096 | airway_hyperreactivity |
| C0005893 | quetelet's_index |
| C0006280 | bronchial_dilating_agents |
| C0007012 | carbon_dioxide_gas |
| C0009450 | infectious_disorder |
| C0010200 | coughs |
| C0011849 | disorder_diabetes_mellitus |
| C0011923 | imaging |
| C0013404 | shortness_of_breath_dyspnea |
| C0013658 | educational_achievements |
| C0013956 | emergency_situation |
| C0015672 | lacking_energy |
| C0018799 | disorder_heart |
| C0018802 | ccf_-_congestive_cardiac_failure |
| C0020538 | hypertensive_vascular_degeneration |
| C0021403 | influenza_vaccine |
| C0021461 | inhaler |
| C0024117 | chronic_obstructive_airway_disease |
| C0027235 | n_isopropylatropine |
| C0027524 | nebulizer |
| C0028778 | obstruction |
| C0029456 | osteoporosis |
| C0032285 | lung_inflammations |
| C0033095 | pressure |
| C0033684 | protein |
| C0034065 | pulm_embolism |
| C0034991 | treatment_rehabilitation |
| C0037369 | smokings |
| C0038317 | steroids |
| C0039231 | rapid_heart_beat |
| C0039985 | chest_x-ray |
| C0040405 | cat |
| C0043096 | weightloss |
| C0043144 | wheeze |
| C0052796 | azithromycin_-_chemical |
| C0073992 | salmeterol |
| C0085639 | falls |
| C0162429 | acquired_deficiency |
| C0184633 | oxygen_therapies |
| C0213771 | tiotropium |
| C0242896 | anticholinergic_agent |
| C0279516 | antibacterial_agent |
| C0453996 | tobacco_smoking_behaviour |
| C1136254 | anti-microbial_agents |
| C1299586 | has_difficulty_doing |
| C1306645 | x-rays |
| C1519384 | history_of_smoking |
| C1522704 | exercise_pain_management |
| C0001927 | albuterol |
| C0010054 | coronary artery disease |
| C0231170 | disability |
| C0034067 | emphysema |
| C0849974 | fev_1 |
| C0082607 | fluticasone |
| C0018802 | heart_failure |
| C0024119 | lung function |
| C0027497 | nausea |
| C0523807 | oxygen_saturation |
| C0038454 | stroke |
| C0040329 | tobacco |
| C4038730 | asthma_copd_overlap_syndrome |
| C0024128 | lung_transplant |
| C0024119 | pulmonary_function_test |
| C0037981 | spirometry |

# **CRT^PFT+^ Classification Model Feature Definitions**

## NLP_everPFTlt70

Create an indicator for whether subject ever had a PFT with spirometry indicating pre-bronchodilator FEV1/FVC ratio <0.7 OR post-bronchodilator FEV1/FVC ratio<0.7.

- Yes=1
- No=0

NLP_everPFTlt70 Extraction Guide for Spirometry:

1. Obtain all spirometry reports which were recorded for the study sample.
2. Assess all unique spirometry reports in the electronic health record performed in < 10 years from extraction date, and generalize extraction code to accommodate each report format (because these may differ across time and by hospital/clinic).
   1. Identify location of relevant FEV1/FVC ratio within the report
      1. If the specific FEV1/FVC ratio is not available, but both FEV1 and FVC individually are available, calculate the FEV1/FVC directly.
3. Extract all historical pre- and post-bronchodilator FEV1/FVC results across all spirometry reports for which these tests were recorded.
4. If any pre- or post-bronchodilator FEV1/FVC < 0.7, assign status as Yes=1. Otherwise, assign No=0.

NLP_everPFTlt70 Extraction Guide for Clinical Notes:

1. Find location of FEV1, FVC, forced expiratory volume at 1, or forced vital capacity
2. Extract a short substring of the note starting at the mention of FEV1/FVC
3. Find the position of the first decimal point after the mention of FEV1/FVC
4. Extract a substring of the numeric values around the decimal point and strip out all other characters

## pftCount

Total count of any kind of pulmonary function test.

## iNotWhite

An indicator for whether the subject is White or Non-White.

- White = 0
- Not White = 1

## smkEver

Smoking history:

- None = 0
- Any (Includes both Current and Former Smokers) = 1.

## patient_dxenct

The total number of encounters with a coded diagnosis.

- Includes all diagnosis codes
- NOT limited to the diagnosis codes used within the phenotype (all phenotypes will have the same value for Patient_dxenct)
- Limit to one occurrence per date
- An inpatient stay that spans multiple days will be counted as one date

## nCOPD_ICD (Number of coded COPD diagnoses)

Create a count of ICD9/10 codes for COPD (**Table 3**)

## nCOPDGTE3_365

Ever diagnosed with 3 or more COPD-related ICD codes in **Table 3** within any rolling time window of 365 days:

- No = 0
- Yes = 1

## ageCOPDt1Specific

Age at **first** ICD code specific to COPD in subsection **Table 3**.

## everdxAtPulmClinic

An indicator for whether subject has ever been diagnosed with a COPD-specific ICD code from the list in **Table 3** at a non-pediatric pulmonary clinic.

- Yes = 1
- No = 0

*Pulmonary clinics at each site will need to be identified prior to extracting this variable.

## nBronchitis

Number of coded Bronchitis diagnoses.

| Feature Type | Code | Name |
| --- | --- | --- |
| Diagnosis (ICD9) | 490* | Bronchitis, not specified as acute or chronic (*includes all codes starting with 490*) |
|  | 491* | Chronic bronchitis (*includes all codes starting with 491*) |
| Diagnosis (ICD10) | J40* | Bronchitis, not specified as acute or chronic (*includes all codes starting with J40*) |
|  | J41* | Simple and mucopurulent chronic bronchitis (*includes all codes starting with J41*) |
|  | J42* | Unspecified chronic bronchitis (*includes all codes starting with J42*) |

## nBronchiectasis

Number of coded Bronchiectasis diagnoses.

| Feature Type | Code | Name |
| --- | --- | --- |
| Diagnosis (ICD9) | 494* | Bronchiectasis *(includes all codes starting with 494)* |
| Diagnosis (ICD10) | J47* | Bronchiectasis (*includes all codes starting with J47*) |

## nmedLAMA

Total count of long acting muscarinic antagonists (LAMAs) from the following list prescribed for treatment of lung diseases in medical record:

| Medication/Rx Ingredient | Brand Names | RXNORM INGREDIENT #* |
| --- | --- | --- |
| Aclidinium | Tudorza | 1303098 |
| Glycopyrrolate | Utibron, Seebri | 4955 |
| Tiotropium | Spiriva, Stiolto | 69120 |
| Umeclidinium | Anoro, Incruse | 1487514 |
| **RXNORM Ingredient #s may not be exhaustive.* | | |

## everTiotropium

An indicator for whether subject has ever been prescribed Tiotropium.

- Yes = 1
- No = 0

## everCOPDmed

An indicator for whether subject has ever been prescribed a COPD medication from the list below:

| Medication/Rx Ingredient | Brand Names | RXNORM INGREDIENT #* |
| --- | --- | --- |
| Aclidinium | Tudorza | 1303098 |
| Arformoterol | Brovana | 1002293 |
| Glycopyrrolate | Utibron, Seebri | 4955 |
| Indacaterol | Arcapta, Utibron | 1114326 |
| Olodaterol | Striverdi, Stiolto | 1546059 |
| Roflumilast | Daliresp | 1091836 |
| Theophylline |  | 10438 |
| Tiotropium | Spiriva, Stiolto | 69120 |
| Umeclidinium | Anoro, Incruse | 1487514 |
| Vilanterol | Breo, Anoro | 1424888, 1487518 |
| Azithromycin |  | 18631 |
| Salmeterol | Serevent | 36117 |
| Formoterol |  | 25255 |
| **RXNORM Ingredient #s may not be exhaustive.* | | |

- Yes = 1
- No = 0

# **Algorithm used to assign ever-smoking status, after free text smoking annotations are extracted from patient EMR.**

This algorithm takes as parameters the list of smoking annotations for one patient and the observation date. It produces one smoking label associated with the observation date. If the observation date is not specified, the current timestamp is assumed. The algorithm produces one of the following labels: "Non-Smoker", "Past Smoker", "Current Smoker" or "No Data".

Internally, the algorithm uses the following set of rules to determine smoking status.

- If no smoking annotation is available (i.e., the list of annotations is empty), "No Data" is returned.
- Otherwise, the algorithm assigns a weight to each of the available smoking labels and selects the one with the highest total weight.
- In case of a tie, the following rules are used to break it: if "Current Smoker" label is tied with "Past Smoker" or "Non-Smoker", "Current Smoker" is selected. Otherwise, if "Past Smoker" is tied with "Non-Smoker", "Past Smoker" is selected. "Non-Smoker" tied with any other label is never selected.

The algorithm uses reciprocal float function to calculate a weight for each smoking annotation, depending on how far away in the past the annotation is from the observation date. Then, it calculates the total weight for each label and selects the label which gets the highest total weight. Currently, the algorithm uses the following function definition:

//reciprocal float function
private double recip(Date d1, Date d2){
    //Assumption: d1 >= d2
    long ms1 = d1.getTime();
    long ms2 = d2.getTime();
    long delta = ms1 - ms2;
    final double coef = 3.16e-11;
    float a = 1f;
    float b = 1f;
    return recip(delta, coef, a, b);
}
    
private double recip(long source, double m, float a, float b){
    //f(source) = a/(m*float(source)+b)
    return a / ((source * m) + b);
}

Here, d1 is the observation timestamp and d2 is the annotation timestamp. With the specified coefficient (3.16e-11), the function will produce 1 when observation timestamp = annotation timestamp, 1/2 when annotation timestamp is 1 year before the observation timestamp, 1/3 when the annotation timestamp is 2 years before the observation timestamp, and so on.

After assigning smoke status, random samples can be manually reviewed to evaluate algorithm performance.

# Model Weights and Thresholds for Alternative COPD Phenotyping Algorithms

**Supplementary Table 4. Model weights and thresholds for alternative COPD phenotyping algorithms developed in the Mass General Brigham Biobank.**

| **Feature** | **CRT-PFT+** | **CRT-PFT-** | **CRT+SAFE** | **SAFE+NLP** |
| --- | --- | --- | --- | --- |
| (Intercept) | -1.871 | -0.851 | -0.755 | 0.395 |
| Age | 0.000 | -0.027 |  |  |
| patient_dxenct | -0.001 | -0.001 |  |  |
| ageCOPDt1Specific | 0.013 | 0.036 | 1.91E-04 |  |
| nBronchitis | -0.009 | -0.062 |  |  |
| nBronchiectasis | -0.008 | -0.046 |  |  |
| nmedAthORcopd | 0.000 | -0.007 |  |  |
| nmedLAMA | 0.017 | 0.049 | 0.002 |  |
| everTiotropium | 0.334 | 0.246 | 0.220 |  |
| everCOPDmed | 0.048 | 0.397 |  |  |
| nCOPD_ICD | 0.013 | 0.073 | 0.001 |  |
| nCOPDGTE3_365 | 0.465 | 0.119 | 0.165 |  |
| everdxAtPulmClinic | 0.056 | 0.352 |  |  |
| smkEver | 0.175 | 0.033 |  |  |
| pftCount | -0.016 | -0.036 |  |  |
| everPFTlt70 | 1.750 |  | 1.878 |  |
| iNotWhite* | -0.239* | -0.501* |  |  |
| iMale | 0.000 | -0.061 |  |  |
| C0001617_adrenal_cortical_hormone |  |  |  | -4.71E-05 |
| C0005893_quetelet's_index |  |  | -0.001 |  |
| C0013956_emergency_situation |  |  |  | -0.002 |
| C0015672_lacking_energy |  |  |  | -0.002 |
| C0018799_disorder_heart |  |  |  | -0.014 |
| C0018802_ccf_-_congestive_cardiac_failure |  |  |  | 1.85E-04 |
| C0021461_inhaler |  |  |  | -2.81E-04 |
| C0024117_chronic_obstructive_airway_disease |  |  |  | 0.004 |
| C0032285_lung_inflammations |  |  | -5.29E-05 |  |
| C0040405_cat |  |  |  | -0.002 |
| C0213771_tiotropium |  |  |  | 3.09E-03 |
| C0453996_tobacco_smoking_behaviour |  |  |  | -5.21E-05 |
| C1136254_anti-microbial_agents |  |  | -3.89E-04 | -0.002 |
| C1299586_has_difficulty_doing |  |  |  | -0.002 |
| C1306645_x-rays |  |  |  | -0.001 |
| **COPD Classification Weight Threshold:** | **0.754** | **0.788** | **0.809** | **0.645** |

* We note that the weights for this feature may reflect disparities in diagnosis of COPD, rather than a feature which is truly predictive of COPD case status, and urge the adoption of the more liberal thresholding scheme which does not make this adjustment for non-white patients.

# **Demographic features of validation dataset from Marshfield Clinic**

| **Features** | **Cases**  **(N=46)** | **Controls**  **(N=54)** |
| --- | --- | --- |
| Female, N (%) | 21 (45.7) | 21 (38.9) |
| Race, N (%) |  |  |
| White | 46 (100.0) | 54 (100.0) |
| Ever Smoker, N (%) | 42 (91.3) | 42 (77.8) |

# **Model-based feature distributions for validation dataset from Marshfield Clinic**

| Marshfield Validation Sample (1=Case, 2=Control, 9=Not classified) | Variable | N | 5th Pctl | Minimum | Median | Mean | 95th Pctl | Maximum |
| --- | --- | --- | --- | --- | --- | --- | --- | --- |
| Case | PATIENT_DXENT NCOPD_ICD NBRONCHITIS NBRONCHIECTASIS NMEDLAMA AGECOPDT1SPECIFIC PFTCOUNT AGE NMEDATHORCOPD NHEARTDXENC | 46 46 46 46 46 46 46 46 46 46 | 144.0 6.0 0.0 0.0 0.0 56.9 1.0 63.7 1.0 14.0 | 102.0 3.0 0.0 0.0 0.0 53.7 0.0 61.4 0.0 2.0 | 400.0 41.0 8.5 0.0 0.0 68.0 2.0 80.8 70.0 91.0 | 447.1 75.6 14.3 0.5 4.5 68.5 3.3 80.3 93.7 120.4 | 972.0 183.0 53.0 5.0 32.0 80.2 9.0 92.3 241.0 322.0 | 1190.0 540.0 69.0 10.0 58.0 89.7 20.0 95.5 331.0 562.0 |
| Not classified/Excluded | PATIENT_DXENT NCOPD_ICD NBRONCHITIS NBRONCHIECTASIS NMEDLAMA AGECOPDT1SPECIFIC PFTCOUNT AGE NMEDATHORCOPD NHEARTDXENC | 54 54 54 54 54 54 54 54 54 54 | 70.0 3.0 0.0 0.0 0.0 49.0 0.0 65.6 0.0 4.0 | 34.0 1.0 0.0 0.0 0.0 39.3 0.0 59.4 0.0 0.0 | 362.0 9.5 3.5 0.0 0.0 70.0 0.0 79.7 29.5 79.0 | 390.7 24.9 6.7 0.5 0.2 70.7 1.1 80.5 45.4 94.1 | 848.0 97.0 22.0 3.0 0.0 91.8 5.0 94.9 174.0 243.0 | 1175.0 108.0 61.0 15.0 9.0 93.8 33.0 98.1 332.0 337.0 |
